# Position specific player load during match-play in a professional football club

**DOI:** 10.1371/journal.pone.0198115

**Published:** 2018-05-24

**Authors:** Ivan Baptista, Dag Johansen, André Seabra, Svein Arne Pettersen

**Affiliations:** 1 School of Sport Sciences, University of Tromsø, the Arctic University of Norway, Tromsø, Norway; 2 Computer Science Department, University of Tromsø, the Arctic University of Norway, Tromsø, Norway; 3 Research Centre in Physical Activity, Health and Leisure (CIAFEL), Faculty of Sport, University of Porto, Porto, Portugal; University of L'Aquila, ITALY

## Abstract

There is a rapid growing body of knowledge regarding physical aspects of a football match due to studies using computer-assisted motion analysis. The present study used time-motion analysis and triaxial-accelerometers to obtain new insights about differences in physical profiles of elite football players across playing-positions. Player performance data in 23 official home matches from a professional football club, during two seasons were collected for analysis. Eighteen players from five different playing positions (central backs: n = 3; full-backs: n = 5; central midfielders: n = 6; wide midfielders: n = 3; and central forwards: n = 4), performing a total of 138 observations. A novel finding was that central backs and central midfielders had significantly lower work-rate in sprints, decelerations and accelerations than full-backs, wide midfielders and central forwards (p<0.001). Furthermore, wide midfielders and full-backs performed significantly more turns (>90°) than central backs. The most common distance covered in high-intensity runs (≥19.8 km·h^−1^) for central backs, central midfielders, wide midfielders and central forwards was 1–5 m, but for full-backs was 6–10 m. This may help coaches in developing individualized training programs to meet the demands of each position in match-play.

## Introduction

To understand physical demands of match-play in football objective data is essential andsuch data could be important for practitioners in designing training programs [1]. Of particular importance is the potential value objective data provide for personalized prescription of training load in a cohort of players following the same overall training regime.

Time motion analysis is commonly used in elite football to analyse player and team performance in training and match as it allows quantification of player running activities and indirect verification of the energetics of match-play [2], creating a rapid growing body of knowledge regarding the physical aspects of football training and match-play [3].

Football has a high-intensity intermittent nature [4], characterised by prolonged intermittent exercise interspersed by periods of maximal or close to maximal effort [5]. Players may be required to repeat sprints, accelerations and turns of short duration interspersed by brief recovery periods over an extended period of time, and these activities have been reported as crucial factors for team performance [6–9].

Previous research has focused on the influence of different factors in the players’ match running profiles, such as the tactical systems [10], possession status [11, 12], competitive standard [13], seasonal fluctuations [14], environment [15], opponent [16] and playing positions [17, 18].

Based on robust findings within the research literature, it is evident that specific playing positions have an influence on total match-load. Midfielders appear to cover the greatest overall distances (~11.5 km) while defenders and forwards cover lower distance (10–10.5 km) [4, 19–21]. Regarding high-intensity runs (HIR), the literature shows that, typically, wide midfielders (WM) and full-backs (FB) display superior HIR profiles [20, 22, 23] and central backs (CB) perform a significantly less amount of time sprinting and running with high intensity compared with other positions [1, 17].

The use of only distance and speed may underestimate the calculation of external player workload since this type of time-motion analysis has neglected some essential and specific movements of football (turns, accelerations, decelerations, etc.) that together appear numerous times during every match and may cause significant physical stress on the players [19, 24].

A previous study, with a Norwegian elite football team [24], combined data from triaxial accelerometer and time-motion analysis and experienced that player load was accumulated in a variety of ways across the different playing positions with accelerations and decelerations contributing 7–10% and 5–7%, respectively. Previous research has shown that players in lateral positions (FB and WM) accelerate more often, whereas CB and central midfielders (CM) decelerate less compared to other positions [24–26].

Therefore, the aims of the present study were to establish and compare the physical demands during official match-play in five different playing positions (CB, FB, CM, WM and central forwards [CF]) in a Norwegian elite football team using time-motion and triaxial-accelerometers.

## Methods

### Subjects and match analysis

With approval from UiT The Arctic University of Norway Institutional Review Board, written informed consent from players and approval from Norwegian Centre for Research Data, data on performance in 23 official home matches from the first team (highest level) in a Norwegian elite football club, during two seasons (2016 and 2017), were collected for analysis. The matches were all played on artificial grass surface (Alfheim Stadium, Tromsø, length = 110m; width = 68m). The sample included 18 players (25.2 ± 4.4 years; 76.2 ± 6.4 kg; 181.6 ± 5.6 cm; in age, body mass and height, respectively) across five different playing positions: CB (n = 3, observations[obs] = 35), FB (n = 5, obs = 34), CM (n = 6, obs = 38), WM (n = 3, obs = 18) and CF (n = 4, obs = 13), making a total of 138 observations. These positions were chosen according to team’s main tactic formation and previous research [8, 18, 20, 24, 26, 27].

Data was analysed only if: (1) players completed the entire match, (2) the player played in the same position during all the match and (3) the team used 4-5-1 or 4-3-3 tactic formations.

To ensure players confidentiality, all data was anonymized before analyses.

### Procedures

A stationary radio-based tracking system (ZXY Sport Tracking System, Trondheim Norway) was used to characterize match activity profiles in the team. Each player wore a specially designed belt, wrapped tightly around the waist, with an electronic sensor system at the player’s lumbar spine [28]. The accuracy and reliability of the system in measuring player movements in elite soccer competitions have been described in more detail in previous studies [26, 28, 29].

### Physical performance variables

Physical parameters analysed included: number of accelerations (acc_counts_), acceleration distance per minute—work-rate—(acc_wr_), number of decelerations (dec_counts_), deceleration work-rate (dec_wr_), HIR work-rate (HIR_wr_), HIR distance (HIR_dist_), sprint work-rate (sprint_wr_), sprint distance (sprint_dist_) and turns.

The following locomotor categories were selected: HIR (≥19.8 km·h^−1^) and sprinting (≥25.2 km·h^−1^). The speed thresholds applied for each locomotor categories are similar to those reported in previous research [16, 20, 24, 26].

According to the ZXY Sport Tracking system, accelerations are defined by four event markers: (1) the start of the acceleration event is marked by the acceleration reaching the minimum limit of 1 m·s ^−2^, (2) the acceleration reaches the acceleration limit of 2 m·s ^−2^, (3) the acceleration remains above the 2 m·s ^−2^ for at least 0.5 seconds and (4) the duration of the acceleration ends when it decreases below the minimum acceleration limit (1 m·s ^−2^).

A turn was defined as a continuous and significant rotation of the body in one direction (derived from gyroscope and compass data). When a rotation in the opposite direction is measured, that will be the end of the previous turn and the start of the next turn. Due to the angle threshold used by ZXY Sport Tracking system only turns ≥90 degrees were analysed.

### Statistical analysis

Descriptive statistics (means and standard deviations) were calculated for the total sample and playing position.

Differences in match performance measures by field position were tested with a one-way analysis of variance (ANOVA). When significance was found, a Bonferroni post-hoc test was performed.

Effect sizes (ES), using Cohen`s *d*, was calculated and interpreted as trivial (≤0.2), small (>0.2–0.6), moderate (>0.6–1.2) and large (>1.2). Significance level was set at 0.05 [30]. Statistical analyses were conducted using SPSS version 24.0.

## Results

### Acceleration and deceleration profiles

There were similar patterns in acc_wr_ and dec_wr_ with CB and CM performing less than FB, WM and CF, with the most significant difference being between CB (3.5 ± 0.7) and CF (5.3 ± 1.0) in dec_wr_ (p<0.001).

In relation to acc_counts_ and dec_counts_ WM presented higher values (76.7 ± 12.1; 86.1 ± 14.7) than CB (64.9 ± 9.7; 61.5 ± 10.8) and CM (65.8 ± 15.6; 71.5 ± 20.6) (p<0.001), respectively.

Furthermore, all positions, except CB, performed less acc_counts_ than dec_counts_ during the entire match (**Table 1**).

**Table 1:** Descriptive statistic (mean and standard deviation) and ANOVA analysis (p-value) of different acceleration parameters analysed according to field position and respective Effect Size (ES) of differences observed.

Variables	Central Backs	Full-backs	Central Midfielders	Wide Midfielders	Central Forwards	p-value	Post-hoc multiple comparisons (p<0.05) | Effect Size
Acc_WR_ (m/min)	3.7 (0.7)	4.4 (0.6)	3.7 (1.2)	4.8 (0.9)	5.1 (1.3)	<0.001	CB<FB (0.25); CB<WM (0.33); CB<CF (0.39); FB>CM (0.26); CM<WM (0.34); CM<CF (0.40)
Acc_COUNTS_ (total)	64.9 (9.7)	71.2 (11.6)	65.8 (15.6)	76.7 (12.1)	71.7 (12.0)	0.008	CB<WM (0.28); CM<WM (0.26)
Dec_WR_ (m/min)	3.5 (0.7)	4.6 (0.7)	4.1 (1.4)	5.2 (0.9)	5.3 (1.0)	<0.001	CB<FB (0.39); CB<WM (0.50); CB<CF (0.48); CM<WM (0.31); CM<CF (0.31)
Dec_COUNTS_ (total)	61.5 (10.8)	73.7 (14.0)	71.5 (20.6)	86.1 (14.7)	80.3 (14.6)	<0.001	CB<FB (0.29); CB<WM (0.47); CB<CF (0.33); CM<WM (0.28)

### HIR and sprint profiles

Differences were observed in HIR_WR_ and Sprint_wr_ between CB and the other positions. CB had the lowest values of all positions in both variables but especially pronounced in Sprint_wr_ (0.9 ± 0.5 m/min) when compared with CF (2.5 ± 1.0 m/min) (p<0.001).

Regarding HIR_dist_, CF presented higher values in 26–30 m than all the other positions, while distances of 36–40 and 46–50 m were covered more times by FB (1.7 ± 1.4; 0.9 ± 1.0). CB (0.8 ± 0.9; 0.2 ± 0.6) were the players with lowest values in these longer distances (36–40 and 46–50). Furthermore, distances of 1–5 m were the distances covered more often by CB, CM, WM and CF, whereas FB had higher values in distances of 6–10 m (**Table 2**).

**Table 2:** Descriptive statistics statistic (mean and standard deviation) and ANOVA analysis (p-value) of different HIR distances and work-rate parameters analysed according to field position and respective Effect Size (ES) of differences observed.

Variables	Central Backs	Full-backs	Central Midfielders	Wide Midfielders	Central Forwards	p-value	Post-hoc multiple comparisons (p<0.05) | Effect Size
HIR_WR_ (m/min)	5.2 (1.6)	8.1 (1.7)	8.0 (3.5)	9.2 (1.8)	9.4 (1.6)	<0.001	CB<FB (0.46); CB<CM (0.46); CB<WM (0.54); CB<CF (0.51)
HIR_DIST_ 1–5 m (counts)	8.2 (2.7)	7.5 (2.5)	9.2 (3.1)	10.3 (2.6)	9.3 (4.2)	0.009	FB<WM (0.27)
HIR_DIST_ 6–10 m (counts)	7.6 (2.2)	8.3 (3.0)	8.2 (3.1)	8.9 (2.4)	8.2 (1.9)	0.591	No sig. differences
HIR_DIST_ 11–15 m (counts)	5.0 (2.7)	6.6 (3.0)	6.3 (3.0)	8.1 (3.0)	6.4 (1.4)	0.008	CB<WM (0.33)
HIR_DIST_ 16–20 m (counts)	4.8 (2.1)	5.0 (2.1)	5.2 (2.6)	5.8 (1.7)	6.0 (2.2)	0.301	No sig. differences
HIR_DIST_ 21–25 m (counts)	2.6 (1.5)	3.7 (1.5)	3.7 (2.1)	4.2 (1.9)	5.2 (1.5)	<0.001	CB<WM (0.28); CB<CF (0.40)
HIR_DIST_ 26–30 m (counts)	1.7 (1.1)	2.7 (1.4)	2.7 (1.8)	2.3 (1.0)	4.3 (1.2)	<0.001	CB<FB (0.26); CB<CF (0.50); FB<CF (0.31); CM<CF (0.33); WM<CF (0.35)
HIR_DIST_ 31–35 m (counts)	1.1 (0.8)	1.7 (1.2)	2.2 (1.6)	3.4 (1.9)	2.8 (2.1)	<0.001	CB<CM (0.24); CB<WM (0.41); CB<CF (0.26); FB<WM (0.30)
HIR_DIST_ 36–40 m (counts)	0.8 (0.9)	1.7 (1.4)	1.2 (1.1)	2.0 (0.8)	1.5 (1.1)	0.001	CB<FB (0.31); CB<WM (0.33)
HIR_DIST_ 41–45 m (counts)	0.6 (0.9)	1.0 (1.1)	1.4 (1.3)	1.1 (1.0)	1.5 (1.0)	0.009	CB<CM (0.29)
HIR_DIST_ 46–50 m (counts)	0.2 (0.6)	0.9 (1.0)	0.8 (0.9)	0.8 (1.1)	1.2 (1.4)	0.007	CB<FB (0.23); CB<CF (0.26)

In relation to sprint_dist_ CB, FB, CM and WM performed higher number of 1–5 m, while CF covered higher number of 6–10 m sprints. (**Table 3**).

**Table 3:** Descriptive statistics statistic (mean and standard deviation) and ANOVA analysis (p-value) of different sprint distances and work-rate parameters analysed according field position and respective Effect Size (ES) of differences observed.

Variables	Central Backs	Full-backs	Central Midfielders	Wide Midfielders	Central Forwards	p-value	Post-hoc multiple comparisons (p<0.05) | Effect Size
Sprint_WR_ (m/min)	0.9 (0.5)	2.0 (0.6)	1.4 (1.0)	1.7 (0.7)	2.5 (1.0)	<0.001	CB<FB (0.49); CB<CM (0.26); CB<WM (0.32); CB<CF (0.55); FB>CM (0.24); CM<CF (0.37)
Sprint_DIST_ 1–5 m (counts)	1.9 (1.2)	3.0 (1.7)	2.3 (1.9)	4.2 (1.7)	3.4 (1.6)	<0.001	CB<WM (0.42); CM<WM (0.35)
Sprint_DIST_ 6–10 m (counts)	1.9 (1.5)	2.9 (1.2)	2.2 (1.8)	3.1 (1.6)	3.6 (2.5)	0.007	CB<CF (0.23)
Sprint_DIST_ 11–15 m (counts)	1.0 (1.0)	2.2 (1.5)	1.5 (1.5)	1.8 (1.1)	2.3 (1.7)	0.008	CB<FB (0.29)
Sprint_DIST_ 16–20 m (counts)	0.7 (0.7)	1.6 (1.2)	1.3 (1.5)	1.3 (0.9)	2.6 (1.6)	<0.001	CB<FB (0.25); CB<CF (0.40); CM<CF (0.28); WM<CF (0.26)
Sprint_DIST_ 21–25 m (counts)	0.4 (0.6)	1.1 (0.9)	0.9 (1.1)	1.0 (0.9)	1.5 (1.1)	0.001	CB<FB (0.29); CB<CF (0.33)
Sprint_DIST_ 26–30 m (counts)	0.3 (0.5)	0.7 (0.7)	0.5 (0.6)	0.5 (0.6)	0.4 (0.5)	0.087	No sig. differences
Sprint_DIST_ 31–35 m (counts)	0.1 (0.3)	0.5 (0.6)	0.3 (0.6)	0.4 (0.7)	1.1 (0.8)	<0.001	CB<CF (0.42); FB<CF (0.26); CM<CF (0.34); WM<CF (0.25)
Sprint_DIST_ 36–40 m (counts)	0.0 (1.7)	0.3 (0.5)	0.2 (0.4)	0.0 (0.0)	0.5 (0.7)	0.001	CB<FB (0.24); CB<CF (0.27); WM<CF (0.26)
Sprint_DIST_ >41 m (counts)	0.3 (0.5)	0.4 (0.8)	0.2 (0.5)	0.4 (0.6)	0.2 (0.4)	0.436	No sig. differences

**Fig 1:**
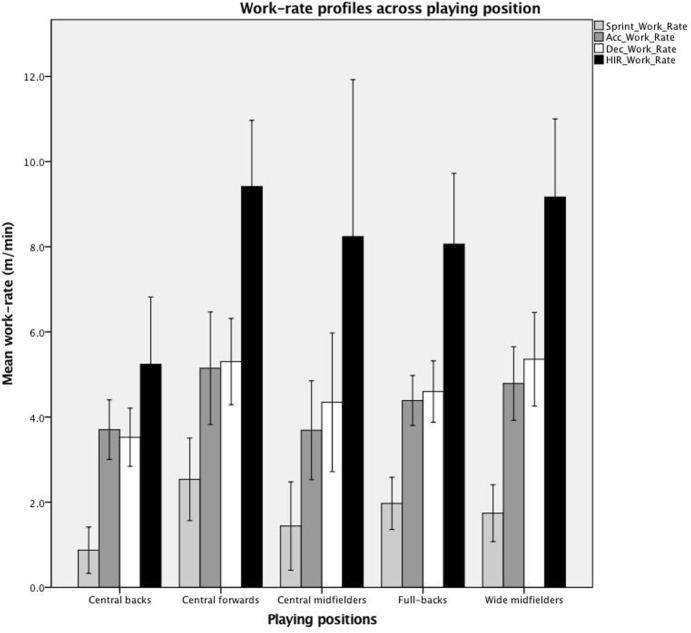
Work-rate profiles across playing position. Mean work-rate in sprints, HIR, acc and dec.

Furthermore, there was a pattern of covariance in the work-rates analysed (acc, dec, HIR and sprint) across playing positions (**Fig 1**).

### Turns

The main outcome was that CB performed less turns per match (32.7 ± 10.1) than FB (41.0 ± 12.1) and WM (42.9 ± 12.3) (p = 0.009).

Moreover, turn angles, 90°-180° were the angles performed more often by all positions, while the turns with the highest angles (271°-360°) were the least common (**Table 4**).

**Table 4:** Descriptive statistics statistic (mean and standard deviation) and ANOVA analysis (p-value) of different parameters of turns analysed according to field position and respective Effect Size (ES) of differences observed.

Variables	Central Backs	Full-backs	Central Midfielders	Wide Midfielders	Central Forwards	p-value	Post-hoc multiple comparisons (p<0.05) | Effect Size
Turns	32.7 (10.1)	41.0 (12.1)	37.0 (12.4)	42.9 (12.3)	41.6 (12.9)	0.009	CB<FB (0.25); CB<WM (0.25)
Turns (90°-180°)	20.3 (6.3)	21.8 (7.2)	20.2 (7.4)	24.2 (6.9)	20.9 (5.7)	0.277	No sig. differences
Turns (181°-270°)	9.8 (5.3)	16.4 (6.1)	13.7 (5.0)	14.9 (6.4)	15.9 (7.8)	<0.001	CB<FB; CB<WM; CB<CF
Turns (271°-360°)	2.3 (1.9)	2.8 (2.1)	3.2 (2.1)	3.6 (1.9)	5.0 (1.9)	0.001	CB<CF; FB<CF

## Discussion

The present study shows that the physical demands in official match-play, in elite football, vary greatly across playing positions. As previously mentioned, a novel finding from this study was that the work-rates in HIR, sprints, accelerations and decelerations change in the same pattern across playing positions. Although further research is needed to verify the correlation between these variables, our results demonstrate that CB and CM had significantly lower work-rate in sprints, accelerations and decelerations than FB, WM and CF with CB also having lower HIR_wr_ than these three playing positions (p<0.001). These findings are in line with the research literature regarding FB covering greater high-intensity and sprinting distances during matches compared to CB. [13, 18, 20, 31].

Previous studies have reported greater distances in HIR and sprint covered by wide players (FB and WM) compared with more central positions (CB, CM and CF) [13, 20, 24, 31], however the present study shows significant higher work-rate for wide positions only in acc, dec and sprints but not in HIR, even though the values for wide positions are slightly, though insignificantly, higher than for central positions (excluding CF). No significant differences were observed between CF and WM in HIR_wr_ which is in line with previous research [18], but in opposition to others [11, 20, 31]. Furthermore, our data show that CF is the most physical demanding position with longer distances covered in HIR, sprints, accelerating and decelerating than the other positions. It has been speculated within the research literature that these differences between wide and more central positions are due to a lack of space for reaching sprinting velocity and the playing style (different roles for different positions) [24, 25, 32]. Taking into consideration the specific context of the club where our data was collected, it seems evident that the style of play (playing many times with low defence and in counter-attacking) had a crucial influence on position’s specific physical demands.

Table 2 illustrates that player position had a significant influence on the different distances covered in HIR. To the best of our knowledge, no previous research has characterized players’ HIR profiles regarding specific distances covered per HIR in official match-play across different playing positions. Our data show that while the most common distance covered in HIR for CB, CM, WM and CF was 1–5 m, for FB it was 6–10 m. An aspect to consider is that we also observed some HIR longer than the ones presented in Table 2 but with no significant differences between positions.

Different patterns appear in sprint_dist_ with CB, FB, CM and WM covering more often shorter distances (1–5 m) in sprint while CF had higher values in longer distances (6–10 m).

Another important finding is that CF and WM accelerated more often compared with players in the other positions, which differs from a previous study with another Norwegian professional football club [24]. However, some similar trends were observed between these studies, with CB being the players who decelerated the least times compared with other playing positions. Furthermore, when comparing our data with results from previous research [4, 24, 25] we observed slightly lower values of acc_counts_ in almost all the positions (CB, FB, CM and WM). The inverse trend was observed in dec_counts_ with all positions presenting higher values in our study, probably due to style of play.

A main finding of the present study refers to the number of turns observed across playing positions. In fact, even though our study has taken into consideration only turns >90° (angle threshold defined by ZXY Sport Tracking), total different values were obtained compared with previous research [17]. One difference is related to the total number of turns per match with our study presenting a mean of ~42 ± 13 to attackers (CF), ~39 ± 13 to midfielders (CM and WM) and ~37 ± 12 to defenders (CB and FB), while previous research [17] presented mean values significantly higher for each position: attackers (~101), midfielders (~107) and defenders (~97) in turns >90°. They observed that midfielders performed significantly fewer turns during a match than defenders and strikers. Our data show that CM did not perform significantly different compared to the other positions while WM performed more turns than CB. These differences may be caused by the different sampling technology used.

Both turns, acceleration and deceleration activities add substantial load in addition to high-intensity running and must be taken into consideration when analysing physical demands of match-play.

It should be noted that different measurement technologies could cause the discrepancy in results between the present study and previous research [5]. Also, different playing styles, cultural and competitive contexts may account for differences observed.

In summary, our data show that speed and distance measures only to some extent predict the physical demands of a football player and that these demands vary greatly across playing positions. Taking into consideration the law of training specificity [33] and the idea that the physical loading of the training session should be individually designed to improve performance and avoid excess of fatigue and overtraining [34] the coaches need a clear view how different playing positions achieve load.

## Practical application

The present results may provide useful and novel insight regarding positional differences in physical profiles of elite football players during match-play. The positional differences in workload and work pattern need to be taken into consideration when designing and implementing training program cycles, according to the team's style of play. As for the team explored in the present study, lateral players should perform some longer sprints ≥ 30 m in normal training weeks to be prepared for these actions that appear during match. Performing sprints in addition to small sided games must be taken into consideration when planning the trainings since small and medium sided games do not provide enough space to elicit these actions.

Apart from providing valuable information to coaches about the activity profiles of different positions, the results may also provide the foundation for a real-time personalization computerized coach toolkit based on our whole-field video analysis system [35] that integrates with positional data in real-time. We are currently developing such a mobile system to customize individual training load to player positions while the practice is unfolding.

## Supporting information

S1 File Data review. (SAV)
